# 

**Published:** 1897-04-10

**Authors:** 


					CADBURY'S ww CADBURY'S
COCOA HP Nl E* COCOA (
" JSinable"-ZaJrJ}^ purity at "resent jR> NO ALKALIES USED.
No. 5^, Yol. XXII. ( ^jfewspaperG SATURDAY,
April 10th, 1897.
[bubscription lcrms,J pRICE TWOPENCE.

				

